# Pediatric Chronic Orofacial Pain: A Narrative Review of Biopsychosocial Associations and Treatment Approaches

**DOI:** 10.3389/fpain.2021.790420

**Published:** 2021-12-20

**Authors:** Linda Sangalli, Robert Gibler, Ian Boggero

**Affiliations:** ^1^Department of Oral Health Science, Division of Orofacial Pain, University of Kentucky, College of Dentistry, Lexington, KY, United States; ^2^Division of Behavioral Medicine and Clinical Psychology, Cincinnati Children's Hospital Medical Center, Cincinnati, OH, United States

**Keywords:** orofacial pain, children, adolescents, psychological comorbidities, familiar factors, headaches, temporomandibular disorder (TMD), pediatric population

## Abstract

Pediatric chronic orofacial pain (OFP) is an umbrella term which refers to pain associated with the hard and soft tissues of the head, face, and neck lasting >3 months in patients younger than 18 years of age. Common chronic pediatric OFP diagnoses include temporomandibular disorder, headaches, and neuropathic pain. Chronic OFP can adversely affect youth's daily functioning and development in many areas of well-being, and may be associated with emotional stress, depression, functional avoidance, and poor sleep, among other negative outcomes. In this mini-review, we will discuss common psychological comorbidities and familial factors that often accompany chronic pediatric OFP conditions. We will also discuss traditional management approaches for pediatric orofacial pain including education, occlusal appliances, and psychological treatments such as relaxation, mindfulness-based interventions, and cognitive-behavioral treatments. Finally, we highlight avenues for future research, as a better understanding of chronic OFP comorbidities in childhood has the potential to prevent long-term pain-related disability in adulthood.

## Introduction

Pediatric orofacial pain (OFP) is an umbrella term which refers to pain associated with hard and soft tissues of the head, face, and neck in patients younger than 18 years of age (1). The most common chronic pediatric OFP complaints include temporomandibular disorder (TMD), headaches, and neuropathic pain (NP) (2). Pain is classified as chronic when it occurs more than 15 days per month, for at least 3 months, and lasts longer than 4 h daily (when present) (3). Compared to adults, youth with chronic OFP may have difficulties in verbalizing symptoms (4), may be poor historians, may experience symptoms that are different from those experienced by adults (5), and may not seek treatment as early or as often (4). Parents and other caregivers play important roles in shaping children's pain experience, and pain in youth affects well-being and functioning in multiple domains. The aims of this mini-review are to describe each of these common chronic pediatric OFP conditions, discuss the roles of psychological, familial and social factors pertinent to conceptualizing the experiences of youth with OFP, and provide a brief overview of evidence-based treatment approaches for children and adolescents with chronic OFP conditions.

## Temporomandibular Disorders

The most frequent chronic pediatric OFP complaint is TMD, a broad term which involves masticatory muscles, the temporomandibular joint (TMJ), and/or their associated structures (1). The prevalence ranges from 4.2 to 68% (6–10), with a recent meta-analysis indicating an average prevalence of 11% (11). Broad variability in prevalence estimates can be attributed to differences in diagnostic criteria (12), varying methods of assessment (13), and diagnostic uncertainty related to use of proxy-reported symptoms (2). Like other pediatric OFP complaints, the prevalence of TMD increases with age, especially in females (4). The most vulnerable pediatric population includes female adolescents, with genetic susceptibility and hormonal fluctuations. Common comorbid conditions with pediatric TMD can include Marfar and Elhers-Danlos syndrome (14), rheumatoid arthritis (15), juvenile idiopathic arthritis (16), psoriatic arthritis, ankylosing spondylitis, and systemic lupus erythematosus, among others (17). The most common signs and symptoms of TMD in pediatric population are articular sounds (popping, clicking, grinding) and masticatory muscle pain (18). Other complaints include limitation and/or asymmetry of mandibular movements, TMJ pain or tenderness, and pain referred to the head, ear or dental structures (18).

Pediatric TMD pain is typically described as constant, dull, and moderate in severity. It is usually aggravated by mandibular function (e.g., talking, chewing, yawning), parafunctional habits (e.g., gum/ice chewing, clenching, grinding, nail/cheek/lip biting), psychological distress (e.g., anxiety, depression, familial distress), and poor ergonomics (e.g., secondary to computer use) (19). In youth, the onset of TMD is usually precipitated by factors such as facial or mandibular trauma (e.g., sports-related jaw injuries) or microtrauma caused by repetitious parafunctional habits (20, 21). Hence, clinicians should always search for extraoral signs indicative of previous trauma (e.g., a scar on the chin).

Youth suffering from TMD frequently experience difficulties in domains of emotional and behavioral functioning compared to their pain-free peers (22, 23), with higher rates of anxiety (24, 25), depression (26), post-traumatic stress disorder (PTSD), fatigue (22), and lower quality of life (22, 27). Research suggests that emotional stress is associated with increased muscle hyperactivity and sleep bruxism (28), which in turn increase the risk of developing TMD (29). Psychosocial factors may play a role in the etiology of TMD (30), and also in the maintenance and long-term prognosis of these conditions (31). Management of psychological distress can mitigate TMD-related signs and symptoms (31) and chronic pain in general (32). Recent longitudinal data suggest that addressing depressive symptoms early in development may be associated with better long-term outcomes for youth with chronic pain conditions (33). Conversely, if not treated, psychological distress associated with TMD pain in adolescence may triple the risk of TMD in young adulthood (34). Thus, interventions that address both pain and psychological distress may be important for improving long-term outcomes in those with pediatric TMD.

## Headaches

The two most common pediatric headaches are tension-type headache (TTH) and migraine, which often co-occur (35). TTH is characterized by a bilateral pressing tightness, with a bandlike distribution around the head or in the occipital region (36, 37). The pain is usually mild to moderate in intensity, higher in late afternoon or evening, and directly related to age and stressors. According to the International Headache Society's International Classification of Headache Disorders (ICHD-III) (36), TTH is classified as episodic (infrequent or frequent) or chronic, depending on the frequency of headaches. Approximately 10.4% (38) to 30.5% (39, 40) suffer from *frequent* headaches, defined as more than one headache episode but <15 headache episodes per month (41). Additionally, 5–20.5% of children experience *chronic* headache (42), defined as headaches occurring more than 15 days per month for at least 3 months (43), although prevalence estimates for chronic headache vary among studies due to differences in methodological and diagnostic criteria (44, 45). Patients with chronic headache are predisposed to develop other comorbid conditions (45). Headache tends to first appear during the school-age years (2), and the prevalence increases throughout childhood and peaks around 11–13 years old (46). Post-pubertal females are at a higher risk for headaches than males (37). The prevalence of youth suffering from headaches has substantially increased over the past 30 years (47, 48), and headache is now considered a major public health problem among high school students (49).

ICHD-III classifies migraine as headache attacks lasting 4–72 h, on <15 days per month, with at least 5 days of migraine per month (36). The overall prevalence of migraine with or without aura (according to the presence of reversible visual and/or sensory and/or speech symptoms without motor weakness for <1 h) in youth varies from 7.7% (44) to 9.1% (37, 50). Conversely, headache attacks occurring on 15 or more days per month for more than 3 months, with at least eight migraine days per month, are classified as *chronic* migraine (36), with a prevalence of 1.5–2% (51). Migraine usually presents as uni- or bilateral throbbing of moderate to severe pain, often located on the forehead (52). In youth, a migraine attack reaches the maximum intensity within 1–2 h and lasts from 2 to 72 h (5). There is evidence that the associated symptoms of migraine vary with age (53) and gender (42). Preschool children most often complain of abdominal pain, vomiting, and fatigue, while school-aged children frequently experience nausea, phonophobia/photophobia, and bilateral frontal pain. In contrast, adolescents commonly experience the typical unilateral temporal headache (53), sometimes preceded by the classical aura (54). However, the difficulty of younger children in describing their symptoms may also explain the difference in reporting the associated aura (55). Because of this difficulty in rating pain and self-reporting typical associated symptoms (56), it is critical to recognize a migraine attack by other signs and symptoms, including pallor, irritability, crying, seeking a dark room to sleep, changes in behavior, loss of appetite, nausea or vomiting, and complains of intolerance to light, noise and exercise (57). Triggers can be challenging to identify, though one third of children report stress, lack of sleep, missing meals, and environmental factors such as changing altitude and barometric pressure, strong odors, computer screens and bright light as triggers (53). Oftentimes, a headache diary may help identify triggering factors and clarify temporal patterns and frequency of attacks. Females more commonly experience the presence of aura, while males more frequently report vomiting and phonophobia (42). Males are affected slightly more than females in early childhood, while an opposite trend is seen following puberty, possibly due to psychological factors, puberty-related hormonal changes (58), and increased susceptibility to stress (59).

TTH and migraine share several common features, and youth who have a positive familial history for these conditions appear to be more susceptible to developing them as well (60). Pediatric patients suffering from headaches may often also experience physical and emotional distress, including stress, anxiety, fatigue, depression, and somatic symptoms (46, 61). They frequently report interference with home and familial activities, social interactions (62), learning difficulties (41), and conduct and inattention-hyperactivity problems (47). In fact, research suggests that impact of headaches on quality of life is similar to that reported by youth with diseases such as arthritis and some cancers (63). Headaches differ from other OFP complaints in several ways. For example, children with headaches report greater sleep disturbances compared to healthy controls (64, 65). Also, one study comparing youth suffering from TTH headache and migraine revealed that children with migraine were twice as likely to suffer from obstructive sleep apnea compared to children with TTH (66), and treating comorbid sleep disorders often leads to improvement of migraine (67) and headache characteristics (68). Moreover, youth suffering from headaches often report a connection between their symptoms and changes in environmental factors, particularly during the academic year (53). Therefore, school-related stress is can be both a triggering factor (69) and a maintaining factor for headache episodes (70).

## Neuropathic Pain

Another chronic pediatric OFP category is neuropathic pain (NP), defined as pain arising as a direct consequence of a lesion or disease affecting the somatosensory system (1). NP is rarely studied systematically in youth because of its relatively low prevalence, which accounts for 6% of children (71). Physical trauma is the most common cause of NP in youth, followed by Fabry disease, chronic human immunodeficiency virus and zoster infections, and complex regional pain syndromes (2). It is characterized by episodes of very brief electric-shock like and abrupt pain (72). The diagnosis is often complicated by the inconclusive clinical examination, which does not confirm any involvement of the corresponding motor or sensory nerve or dermatome distribution.

Youth with chronic NP often report significant mood disturbances, high levels of pain catastrophizing, limitations in daily activities, and diminished quality of life and physical functioning (73). Moderate to severe levels of anxiety and depression appear to be higher in females than in males with NP (74). Youth with NP and their parents report similarly low health-related quality of life. However, research suggests that parents who score in higher elevated ranges on measures of anxiety and depression also tend to catastrophize about their child's pain *even more* than their children do themselves, underscoring the influences of parental beliefs about child pain on overall family functioning (75).

## Family and Social Factors in Pediatric OFP

Parents play crucial roles in shaping their child's pain experience across ages and stages of development (76). Chronic pain of any nature in childhood is associated with substantial familial burden across multiple domains of daily living (77). Parents often understandably feel overwhelmed by juggling the many demands that caring for a child with chronic pain often requires, including attending medical appointments, adjusting to activity restrictions, managing school-related challenges, and addressing mental health concerns. Consequently, it is perhaps not surprising that compared to their pain-free peers, children and adolescents with chronic pain generally report higher levels of family conflict (78). Among adolescents with chronic headache, research suggests poorer overall family functioning—characterized by greater conflict and restriction of adolescent autonomy—is associated with greater child functional disability and depressive symptoms (79).

While familial conflict can be predictive of poorer pain-related and emotional outcomes in youth, research has also identified *specific* parental cognitive and behavioral factors that are associated with higher disability in the child. Parental pain catastrophizing, or the parent's tendency to magnify, ruminate or feel helpless about pain and its perceived consequences, is robustly associated with poorer child functioning (80, 81). Research among youth with headaches has shown that parents who engage in higher levels of pain catastrophizing rate their children's pain as more severe (82). Furthermore, parents who tend to catastrophize about their child pain may also engage in more solicitous and overprotective parenting behavior in order to mitigate their child's distress as well as their own (83, 84). Though well-intentioned, these behaviors limit adaptive coping and are associated with poorer child functioning (85).

Orofacial pain also presents a number of social challenges for youth. Children and adolescents with headaches report substantially lower health-related quality of life compared to healthy controls (63), which may be related to the *anticipation* of a starting or worsening headache or worry about the possible consequences of missing social, athletic, or academic activities due to pain (86, 87). In contrast, adolescents with TMD pain face unique challenges related to deficits in jaw function that may affect smiling, eating, and talking (88). One qualitative study of youth with JIA and co-occurring TMD conducted by Leksell et al. (89) revealed that parents often worry considerably about the types of foods they can serve their child if their jaw pain is severe. Furthermore, parents of youth with TMDs may elect to send certain foods with their children out of concern that available food may be unsuitable for them (e.g., at a friend's home). Though speculative given the paucity of research examining the social ramifications of pediatric TMD pain, it is certainly conceivable that making these types of adjustments to one's daily life and experiencing difficulties with basic social functions could be embarrassing, anxiety-provoking, and stigmatizing for children and adolescents. Researchers have recently made a call to action to more carefully delineate the social consequences of TMDs in youth and adult populations given the considerable stigma associated with these conditions (90).

## Treatment of Pediatric OFP

In chronic pediatric OFP populations, multidisciplinary treatment that combines orofacial pain specialists, pharmacotherapy, psychology, physiotherapy (56), and lifestyle modifications is often recommended (53). Treatment often starts with evaluation from an orofacial pain-trained dentist or a neurologist, and includes education of both the patient and the family about the treatable nature of the condition. In clinical settings, pediatric patients with headaches are often encouraged to avoid triggering factors, such as an overloaded daily routine (53), maintain regular food and fluid intake (91), obtain sufficient sleep (92), and engage in regular physical exercise (91).

Although maintaining healthy lifestyle habits is crucial for pain management, psychological interventions that help youth develop and implement *pain coping skills* such as relaxation training and cognitive restructuring can make a significant difference. Cognitive-behavioral therapy (CBT) is a skills-focused intervention that has shown to be effective for treating a range of pediatric chronic pain conditions (93). Recent evidence suggests that participation in CBT may even lead to changes in functional connectivity in brain regions involved in nociceptive processing and emotional regulation (64, 94). Treatment of TMD stresses the importance of habit reversal for repetitive daytime parafunctional activities and overuse of masticatory muscles. These interventions frequently involve psychologists and other behavioral health providers (95), and may include an occlusal appliance for nighttime use.

Regardless of the OFP condition, it is important to establish to what extent psychological factors like stress, anxiety, or depression may be playing a role in exacerbating and maintaining pain and associated symptoms. There is evidence that non-pharmacological approaches may be more effective in treating children suffering from chronic headaches compared to adults (5, 93). These interventions can include CBT, relaxation, biofeedback, psychotherapy (47), physical self-regulation (96), or a combination of behavioral and exercise-based interventions (97). These non-pharmacological interventions are intended to help patients learn skills needed to cope with chronic pain (56), mitigate pain-related disability, and reduce psychological distress (98). These approaches can either replace or be used in combination with pharmacological interventions, which instead might take weeks to months for a significant reduction in pain, especially with headache (53). The same behavioral approaches can be used for TMD treatments, although less efficacious than occlusal appliances (99).

Pharmacological approaches for pediatric OFP have often been translated from studies conducted on adults, without a careful assessment prior to implementation. Overall, they differ upon diagnosis and existing associated comorbidities. Chronic headaches can be treated with daily preventive medications, including tricyclic antidepressants (TCA, such as amitriptyline), anticonvulsant (topiramate) or beta-blockers (propranolol) (53). When comorbid mood disorders are present, selective serotonin reuptake inhibitors (SSRI) or selective norepinephrine reuptake inhibitors (SNRI) might be considered (53). However, there is evidence that commonly prescribed medications such as amitriptyline and topiramate for the management of headaches in children perform equally well to placebo (100), and non-pharmacological interventions such as CBT are effective in reducing headache days and headache-related disability among youth (101).

As for TMD, the management can require a trial of clock-regulated non-steroidal anti-inflammatory drugs (NSAID, as meloxicam or piroxicam), muscle relaxers or anxiolytic agents to disrupt the chronic musculoskeletal cycle of muscle fatigue and energy crisis (95). Data regarding pediatric NP in the orofacial area are limited and suggest the use of antiepileptics (gabapentin), TCA (amitriptyline), or anticonvulsants (oxcarbazepine, carbamazepine) (102). However, pharmacotherapy is not exempt from side effects, including sedation, sleep problems, and weight gain, all of which might be a significant concern in teenagers (53). Moreover, it is important to warn the patient and the family about the risk of analgesic rebound headache, thus encouraging to limit analgesic use to no more than twice per week (103).

## Discussion and Future Directions in Pediatric OFP

Despite the prevalence and impact of chronic pediatric OFP conditions, much remains to be learned about the etiology, biopsychosocial comorbidities, and long-term prognosis. Important areas for future research include, but are not limited to, chronic pain mechanisms and maintenance among pediatric patients, the potential role of neuronal plasticity in the chronicity of pediatric OFP conditions, and how psychosocial functioning interacts with pain pathways to promote disability or resilience. Randomized clinical trials on pharmacological approaches specifically conducted on pediatric population are warranted. Future research should aim to validate diagnostic criteria specifically for youth as clinical features, chronicity, frequency and triggering factors appear to be different from adults. More longitudinal data are needed to fully understand how familial and social factors impact youth with orofacial pain. Investigating the expectation of the youth on treatment outcomes is also an important area for future research. Finally, more work needs to be done in developing more effective multidisciplinary treatments for these conditions, including behavioral modification and coping strategy interventions, not only directed to the patients themselves but also to their families. Assessing the efficacy of these multidisciplinary treatments may also predict a long-term prognosis. It is likely that advances in each of these areas will eventually lead to a better understanding of pediatric OFP conditions, to the complex multidisciplinary contributions, and to improvements in the treatment options that are already currently available.

## Author Contributions

LS, RG, and IB gave substantial contribution to the conception and the design of the study, drafted the article, and revised it critically. All authors have read and approved the final version of the manuscript.

## Funding

IB was supported as a DREAMS Scholar at the University of Kentucky. This award was supported by the National Institutes of Health (NIH) National Center for Advancing Translational Sciences through grant number UL1TR001998. The content is solely the responsibility of the authors and does not necessarily represent the official views of the NIH.

## Conflict of Interest

The authors declare that the research was conducted in the absence of any commercial or financial relationships that could be construed as a potential conflict of interest.

## Publisher's Note

All claims expressed in this article are solely those of the authors and do not necessarily represent those of their affiliated organizations, or those of the publisher, the editors and the reviewers. Any product that may be evaluated in this article, or claim that may be made by its manufacturer, is not guaranteed or endorsed by the publisher.
